# CALHM1 and its polymorphism P86L differentially control Ca^2+^ homeostasis, mitogen‐activated protein kinase signaling, and cell vulnerability upon exposure to amyloid β

**DOI:** 10.1111/acel.12403

**Published:** 2015-09-29

**Authors:** Ana José Moreno‐Ortega, Izaskun Buendia, Lamia Mouhid, Javier Egea, Susana Lucea, Ana Ruiz‐Nuño, Manuela G. López, María F. Cano‐Abad

**Affiliations:** ^1^ Servicio de Farmacología ClínicaInstituto de Investigación SanitariaHospital Universitario de la PrincesaMadridSpain; ^2^ Instituto Teófilo HernandoUniversidad Autónoma de MadridMadridSpain; ^3^ Departamento de Farmacología y TerapéuticaFacultad de MedicinaUniversidad Autónoma de MadridMadridSpain

**Keywords:** Alzheimer's disease, Ca^2+^ channel CALHM1, CREB, Ca^2+^ homeostasis, caspases, early apoptosis

## Abstract

The mutated form of the Ca^2+^ channel CALHM1 (Ca^2+^ homeostasis modulator 1), P86L‐CALHM1, has been correlated with early onset of Alzheimer's disease (AD). P86L‐CALHM1 increases production of amyloid beta (Aβ) upon extracellular Ca^2+^ removal and its subsequent addback. The aim of this study was to investigate the effect of the overexpression of CALHM1 and P86L‐CALHM, upon Aβ treatment, on the following: (i) the intracellular Ca^2+^ signal pathway; (ii) cell survival proteins ERK1/2 and Ca^2+^/cAMP response element binding (CREB); and (iii) cell vulnerability after treatment with Aβ. Using aequorins to measure the effect of nuclear Ca^2+^ concentrations ([Ca^2+^]_n_) and cytosolic Ca^2+^ concentrations ([Ca^2+^]_c_) on Ca^2+^ entry conditions, we observed that baseline [Ca^2+^]_n_ was higher in CALHM1 and P86L‐CALHM1 cells than in control cells. Moreover, exposure to Aβ affected [Ca^2+^]_c_ levels in HeLa cells overexpressing CALHM1 and P86L‐CALHM1 compared with control cells. Treatment with Aβ elicited a significant decrease in the cell survival proteins p‐ERK and p‐CREB, an increase in the activity of caspases 3 and 7, and more frequent cell death by inducing early apoptosis in P86L‐CALHM1‐overexpressing cells than in CALHM1 or control cells. These results suggest that in the presence of Aβ, P86L‐CALHM1 shifts the balance between neurodegeneration and neuronal survival toward the stimulation of pro‐cytotoxic pathways, thus potentially contributing to its deleterious effects in AD.

## Introduction

Alzheimer's disease (AD) is clinically characterized by progressive cognitive impairment that is believed to result from synaptic dysfunction and neurodegeneration initiated by the aggregated form of amyloid beta (Aβ) peptide (Hardy & Selkoe, 2002). Accumulated evidence suggests that AD is also linked to an imbalance of intracellular Ca^2+^ homeostasis (Bezprozvanny & Mattson, 2008; Green & LaFerla, 2008; Marambaud *et al*., 2009; Fernandez‐Morales *et al*., 2012), because Ca^2+^ plays a critical role in maintaining cell survival; for example, a mild elevation of [Ca^2+^]_c_ promotes neuronal survival and plasticity, whereas more pronounced elevations can cause neurotoxicity (Berridge *et al*., 1998; Cano‐Abad *et al*., 2001). Thus, alterations in Ca^2+^ homeostatic mechanisms associated with aging, mutations in amyloid precursor protein (APP) and presenilins, and dysfunctional Ca^2+^ fluxes at the endoplasmic reticulum (ER) can promote neuronal cell death (Bezprozvanny & Mattson, 2008).

Although data from the literature indicate that neuronal death in AD is related to the action of Aβ on intracellular Ca^2+^ dyshomeostasis, little is known about the role of the novel Ca^2+^ channel, calcium homeostasis modulator 1 (CALHM1), in the disease. CALHM1 is expressed in all brain regions and neuronal cells, at the ER, and in the plasma membrane. CALHM1 generates Ca^2+^‐selective cation currents in the plasma membrane. It has also been shown to form a novel Ca^2+^‐permeable ion channel, whose gating is allosterically regulated by both membrane voltage and extracellular Ca^2+^ concentration; in addition, CALHM1 is insensitive to classic selective blockers of voltage‐gated Ca^2+^ channels, although it is inhibited by nonselective and inorganic Ca^2+^ channel blockers such as Co^2+^ (Dreses‐Werringloer *et al*., 2008; Moreno‐Ortega *et al*., 2010; Ma *et al*., 2012). But recently we described that CAHM1 is blocked by CGP37157 (Moreno‐Ortega *et al*., 2015).

A polymorphism of CALHM1, P86L‐CALHM1, which results in a proline to leucine substitution at codon 86, has been associated with early onset of sporadic AD (Dreses‐Werringloer *et al*., 2008); however, this association remains controversial. Thus, while some studies have shown a significant correlation (Boada *et al*., 2010; Cui *et al*., 2010), others have failed to find such an association (Bertram *et al*., 2008). While it is accepted that P86L‐CALHM1 is not a genetic risk factor for the development of AD, a meta‐analysis has shown that this polymorphism modulates the age of disease onset (Lambert *et al*., 2010). Transient expression of the P86L‐CALHM1 channel promotes accumulation of Aβ by altering membrane permeability to Ca^2+^ and, consequently, promotes an increase in [Ca^2+^]_c_ (Dreses‐Werringloer *et al*., 2008). However, evidence implicating a role for Aβ‐induced disruption of Ca^2+^ homeostasis linked to CALHM1 or P86L‐CALHM1 and the activation of cell death signaling pathways has not been reported.

Selective neuronal vulnerability is a feature of a number of neurodegenerative diseases, but the processes that target specific neurons for death while allowing others to remain healthy are unclear. The differential activation of an internal death program in vulnerable neurons has been proposed as a mechanism to explain the selective death of neurons (Schreiber & Baudry, 1995). However, it is equally likely that specific neuronal populations contain an intrinsic survival mechanism. The presence and/or activity of such a pathway in various cell types could partly explain their varying sensitivities to detrimental brain insults. Several studies have recently implicated the transcription factor c‐AMP response element‐binding protein (CREB) as a possible regulator of a general survival program in neurons. CREB can be activated by various kinases in response to electrical activity, neurotransmitters, hormones, and neurotrophins, thus promoting the expression of many genes that contain cAMP response elements (Finkbeiner *et al*., 1997; Hardingham & Bading, 1998). CREB also plays a central role in memory formation (West *et al*., 2001). The transcriptional activation of CREB is crucially dependent on phosphorylation of Ser133 by kinases such as Ca^2+^/calmodulin kinase (CaMK), ras/mitogen‐activated protein kinase (MAPK), ERK1/2 (Wu *et al*., 2001), and protein kinases A and C (Hardingham *et al*., 1999). Extracellular signal‐regulated kinases (ERKs) are key genes in activating survival pathways (Roskoski, 2012), and their transient activation plays an important role in memory‐related processes (Costa & Silva, 2002).

As Ca^2+^ dyshomeostasis is found in AD and P86L‐CALHM1 is considered a risk factor for AD, we investigated how native CALHM1 and P86L‐CALHM1 could contribute to Ca^2+^ homeostasis, survival signaling pathways (namely, ERK and the transcription factor CREB), and cell survival at baseline or after treatment with Aβ. We used transfected HeLa cells with the empty vector (control) and cells transfected with vectors including CALHM1 and P86L‐CALHM1 to study the kinetics of the changes of [Ca^2+^]_c_ and [Ca^2+^]_n_ generated by reintroduction of Ca^2+^ and treatment with Aβ. We also analyzed ERK, CREB activation, and apoptosis pathways upon exposure to Aβ. Our results indicate that P86L‐CALHM1 could contribute to neuronal vulnerability by affecting cytosolic and nuclear Ca^2+^ homeostatic mechanisms and survival signaling pathways.

## Results

### Effect of CALHM1 and P86L‐CALHM1 overexpression on the nuclear concentration of Ca^2+^

Several authors have investigated the participation of CALHM1 expression in different Ca^2+^ compartments such as cytosol (Dreses‐Werringloer *et al*., 2008; Moreno‐Ortega *et al*., 2010; Ma *et al*., 2012), mitochondria ([Ca^2+^]_mt_) (Moreno‐Ortega *et al*., 2010), and ER ([Ca^2+^]_ER_) (Gallego‐Sandin *et al*., 2011). However, the regulation of nuclear Ca^2+^ homeostasis by CALHM1 and P86L‐CALHM1 has not yet been described.

Because CALHM1 is anchored to the ER membrane (Dreses‐Werringloer *et al*., 2008) and the ER membrane constitutes the nuclear envelope, we hypothesized that upon CALHM1 opening, and the channel could be releasing Ca^2+^ from the ER into the nucleus. Furthermore, variations in the [Ca^2+^]_c_ can promote changes in [Ca^2+^]_n_ that could regulate cellular functions ranging from proliferation to cell death (Alonso *et al*., 2011). Therefore, we used nuclear‐targeted aequorin (nu_AEQ) to explore whether CALHM1 or P86L‐CALHM1 overexpression could promote changes in [Ca^2+^]_n_ upon reintroduction of Ca^2+^.

**Figure 1:**
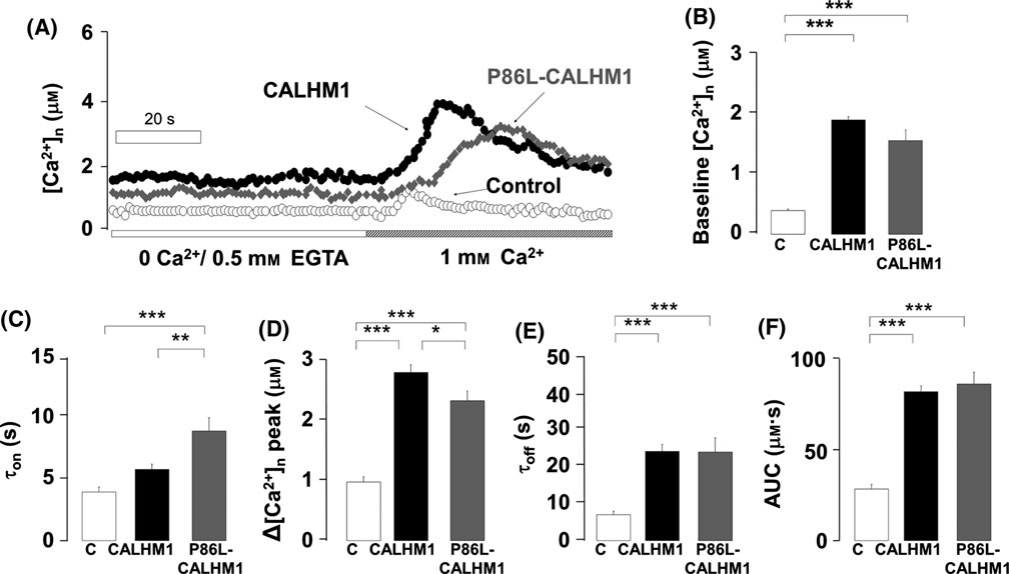
Kinetics of the nuclear Ca^2+^ transients ([Ca^2+^]_n_) measured using aequorin targeting the nucleus. (A) Typical traces of the time course of [Ca^2+^]_n_ elevation elicited during the time period is indicated. Ca^2+^ was reintroduced as indicated on the bottom horizontal bar. Data are represented as follows: (B) baseline [Ca^2+^]_n_, (C) time constant for activation (τ_on_), (D) peak [Ca^2+^]_n_ transient amplitude, (E) time constant for inactivation (τ_off_), and (F) area under the curve (AUC) of the transients in cells overexpressing the empty vector (C), CALHM1, or P86L‐CALHM1. Bar graphs of B–F were computed with pooled data from 20 experiments (control), 34 experiments (CALHM1), and 29 experiments (P86L‐CALHM1) performed with cells from 10 different cultures and according to protocols such as those shown in A. Data are expressed as mean ± SEM. anova post hoc Bonferroni, *P < 0.05; **P < 0.01; ***P < 0.001.

Cells transfected with nu_AEQ were initially perfused with a 0 Ca^2+^/EGTA solution for 2 min. This solution was then switched to another one containing 1 mm Ca^2+^. Figure 1A shows that the [Ca^2+^]_n_ was stable at around 0.38 μm in control cells in 0 Ca^2+^/EGTA; in CALHM1 and P86L‐CALHM1 cells, [Ca^2+^]_n_ was quite stable at 1.83 and 1.5 μm, respectively. Upon reintroduction of 1 mm Ca^2+^, [Ca^2+^]_n_ rose to a peak at 0.97 ± 0.09 μm and then decayed to near baseline values, indicating inactivation of the constitutive capacitative Ca^2+^ entry channel of the control HeLa cells. The kinetics of the transient [Ca^2+^]_n_ in CALHM1‐overexpressing cells were considerably different from those of the control; the activation rate was significantly slower and peaked at 2.79 ± 0.13 μm before slowly decaying to a stable plateau at around 1.5 μm. In P86L‐CALHM1‐overexpressing cells, the transient [Ca^2+^]_n_ developed much more slowly, reaching a peak at 2.32 ± 0.16 μm and stabilizing as a plateau, with little decay.

Quantitative averaged data from 20, 34, and 29 experiments for control, CALHM1, and P86L‐CALHM1 cells, respectively, show a 4.86‐fold increase over baseline [Ca^2+^]_n_ in CALHM1 cells and 3.98‐fold increase for P86L‐CALHM1 cells, with respect to the control cells (Fig. 1B). In addition, the kinetics of the [Ca^2+^]_n_ transients differed between the three cell types. For instance, the time constant for the rate of the transient rise (τ_on_) was 1.47‐fold and 2.27‐fold higher in CALHM1 and P86L‐CALHM1 cells, respectively, than in control cells (Fig. 1C), suggesting slower activation of the [Ca^2+^]_n_ signal. Moreover, the peak heights were 2.86‐fold and 2.34‐fold greater (Fig. 1D). The rate of signal decay was considerably slower in CALHM1 cells (τ_off_ 3.46‐fold higher) and in P86L‐CALHM1 cells (τ_off_ 3.41‐fold higher), with respect to control cells (Fig. 1E). Finally, we calculated the area under the curve (AUC) of each transient as a reflection of the total [Ca^2+^]_n_, considering the net rise in [Ca^2+^]_n_ from baseline for each cell type: we observed that it was 2.87‐fold higher in CALHM1 and 3.03‐fold higher in P86L‐CALHM1 cells than in controls (Fig. 1F).

### CALHM1 and P86L‐CALHM1 overexpression and Ca^2+^ release at nucleoplasma regions

**Figure 2:**
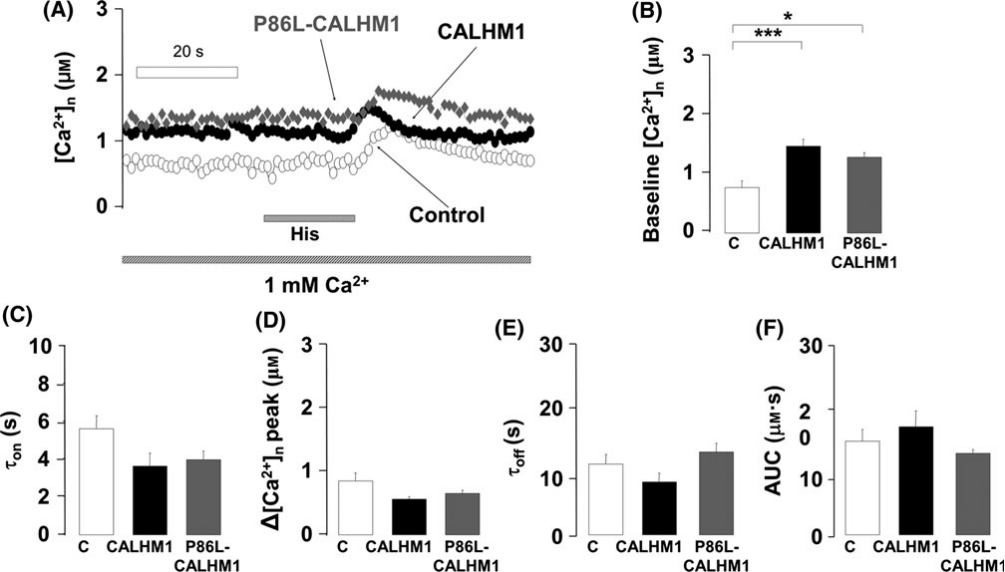
Kinetics of the [Ca^2+^]_n_ transients elicited by histamine in control, CALHM1, and P86L‐CALHM1 cells. (A) Typical traces of the time course of [Ca^2+^]_n_ elicited by 100 μm histamine, administered as indicated on the bottom bar. Data are analyzed as follows: (B) baseline [Ca^2+^]_n_, (C) time constant for activation (τ_on_), (D) peak [Ca^2+^]_n_ transient amplitude, (E) time constant for inactivation (τ_off_), and (F) area under the curve (AUC) of the transients in cells expressing the empty vector (C), CALHM1, or P86L‐CALHM1. Bar graphs of B–F were computed with pooled data from 16 experiments performed with cells from seven different cultures and according to the protocols shown in the A. Data are expressed as mean ± SEM. anova post hoc Bonferroni, *P < 0.05; ***P < 0.01.

Activation of inositol 1,4,5‐trisphosphate receptors (InsP_3_R) is a key mechanism of Ca^2+^ entry into the nucleus. As the ER membrane forms part of the nuclear envelope and CALHM1 is anchored to the ER, we explored whether CALHM1 or P86L‐CALHM1 overexpression could affect the kinetics of the [Ca^2+^]_n_ transients elicited by indirect InsP_3_R activation by histamine. We first perfused cells with a Ca^2+^ solution (1 mm) for 2 min and replaced this solution with another one containing 100 μm of histamine for 15 s. Figure 2A shows three superimposed typical traces on the [Ca^2+^]_n_ variations elicited by histamine‐InsP_3_R stimulation. Once more, baseline [Ca^2+^]_n_ was higher in CALHM1 and P86L‐CALHM1 cells than in control (Fig. 2B). As far as the histamine‐elicited transients was concerned, no significant changes were observed in the τ_on_, peak [Ca^2+^]_n_, τ_off_, or AUC between the three cell types (Figs. 2C–F). One interpretation of these results could be that slow inactivation of InsP_3_R channels occurs upon Ca^2+^ leak through CALHM1 and P86L‐CALHM1, which would in turn slower Ca^2+^ release into the nucleus owing to InsP_3_R inactivation by Ca^2+^.

### Effects of Aβ on cytosolic Ca^2+^ signaling in cells overexpressing CALHM1 and P86L‐CALHM1

Previous results gave rise to the hypothesis that CALHM1 could behave as a leak channel regulating changes in the kinetics of nuclear [Ca^2+^]_n_ changes and, in so doing regulates Aβ/APP ratio levels in a Ca^2+^‐dependent manner (Dreses‐Werringloer *et al*., 2008). However, the influence of extracellular Aβ on Ca^2+^ homeostasis in CALHM1‐ and P86L‐CALHM1‐overexpressing cells has not been investigated to date. To address this issue, we performed experiments to measure changes in [Ca^2+^]_c_ occurring during acute Aβ treatment. To reveal possible changes in the rate of Ca^2+^ entry through CALHM1 or P86L‐CALHM1 channels, [Ca^2+^]_c_ was measured under the channel activating form, that is removal of extracellular Ca^2+^ (0 Ca^2+^/EGTA) and its subsequent addback (1 mm Ca^2+^) in the absence or presence of Aβ.

**Figure 3:**
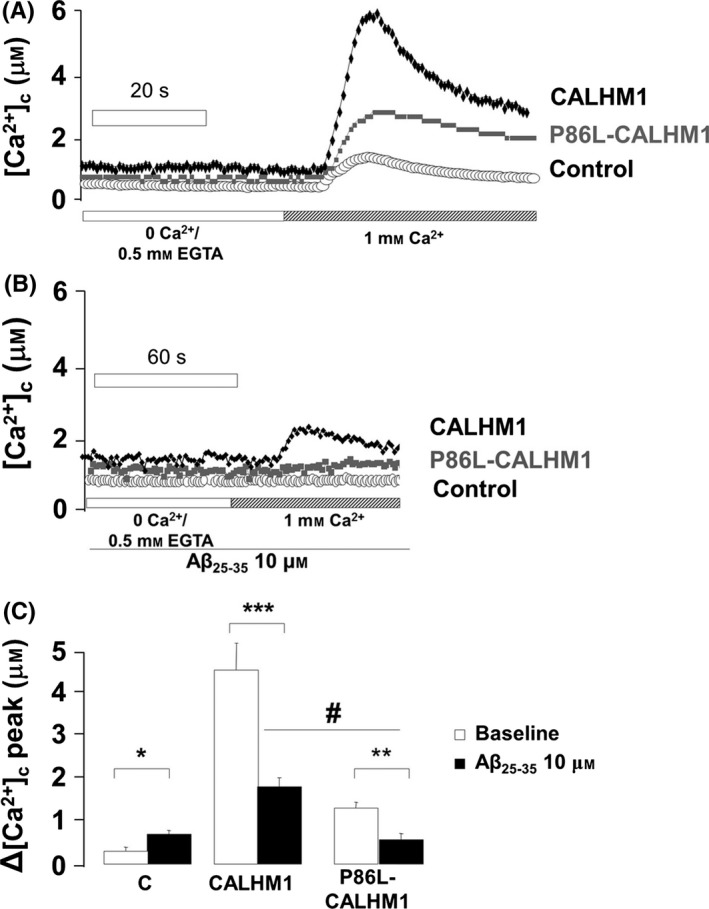
Signaling of cytosolic Ca^2+^ concentrations ([Ca^2+^]_c_) elicited by perfusion with Aβ in HeLa cells overexpressing control, CALHM1, and P86L‐CALHM1. (A) Example of typical traces of [Ca^2+^]_c_ elicited by an addback Ca^2+^ protocol in HeLa cells transfected with the empty vector (control), CALHM1, or P86L‐CALHM1. (B) Experiment performed as in A, but in cells continuously perfused with amyloid β_25–35_ peptide (Aβ_25–35_) at 10 μm. (C) Δ[Ca^2+^]_c_ of the peak of the transient amplitude of the Ca^2+^ entry elicited by the addback protocol. Pooled data are expressed as mean ± SEM from at least seven experiments performed with three different batches of cells. anova post hoc Dunnet, *P < 0.05; **P < 0.01; ***P < 0.001 compared with non‐Aβ control. One‐tail *t‐*test, ^#^ *P* < 0.05 compared CALHM1 versus P86L‐CALHM1.

In the absence of Aβ, the addback of Ca^2+^ elicited a significant increase in the [Ca^2+^]_c_, reaching 4.34 and 1.25 μm in CALHM1 and P86L‐CALHM1, respectively (Fig. 3A). In the presence of Aβ_25‐35_ (10 μm) and 1 mm extracellular Ca^2+^, slight oscillations in baseline [Ca^2+^]_c_ in both CALHM1 and P86L‐CALHM1 cells were detected (data not shown). Extracellular Ca^2+^ was then withdrawn and this protocol repeated in the presence of Aβ; Ca^2+^ entry was significantly reduced in CALHM1‐ or P86L‐CALHM1‐overexpressing cells (Fig. 3B). Pooled data show that in CALHM1 cells, peak [Ca^2+^]_c_ was reduced by 40.32%, from 4.34 μm (no Aβ) to 1.75 μm (plus Aβ), whereas in P86L‐CALHM1 cells, the peak was reduced by 44% from 1.25 to 0.55 μm. In control cells, [Ca^2+^]_c_ changes were mild and similar in the presence or absence of Aβ (0.67 and 0.3 μm, respectively) (Fig. 3C). These modifications seem to be specific for the toxic form of Aβ since the scramble sequence of βA_25–35_ did not afford significant modifications in the [Ca^2+^]_c_ (data not shown).

### Vulnerability of CALHM1‐ and P86L‐CALHM1‐overexpressing cells to different cytotoxic stimuli

**Figure 4:**
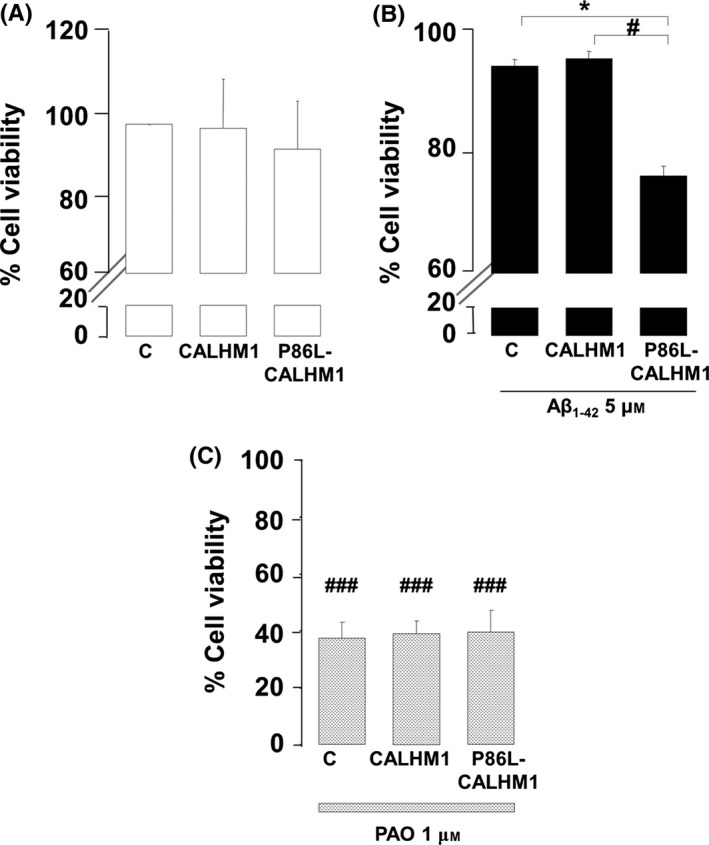
Cell vulnerability upon treatment with Aβ_1–42_ and phenylarsine oxide. The MTT assay was performed to test cell viability in control (C), CALHM1‐, or P86L‐CALHM1‐overexpressing HeLa cells. (A) Baseline viability. (B) Viability after 24 h of treatment with 5 μm of a mixture of protofibrils and oligomers of 1–42 of Aβ (Aβ_1–42_). (C) Viability after 24 h of treatment with 1 μm phenylarsine oxide (PAO). Triplicate measurements were obtained from four different cultures. Data are expressed as mean ± SEM. anova post hoc Bonferroni, *P < 0.05 compared with control cells treated with Aβ. anova post hoc Dunnet, ^#^ *P* < 0.01; ^###^ *P* < 0.001, compared with untreated cells.

No significant baseline cell death was observed in HeLa cells transiently expressing the empty vector (control), CALHM1, or P86L‐CALHM1 (Fig. 4A). When both types were incubated with oligomers of Aβ_1–42_, 5 μm for 24 h, only P86L‐CALHM1‐overexpressing cells showed significant cell toxicity (24% of cell death) compared with cells overexpressing the empty vector or the wild‐type channel (Fig. 4B).

We also evaluated vulnerability to oxidative stress stimuli using phenylarsine oxide (PAO), which causes oxidative stress via a mitochondria‐dependent mechanism (Vay *et al*., 2009). PAO reduced cell viability in all three cell types (Fig. 4C). Therefore, cells expressing P86L‐CALHM1 did not show higher vulnerability to oxidative stress, in contrast to the observations with Aβ treatment.

### Activation of apoptosis in CALHM1‐ and P86L‐CALHM1‐overexpressing cells upon Aβ exposure

**Figure 5:**
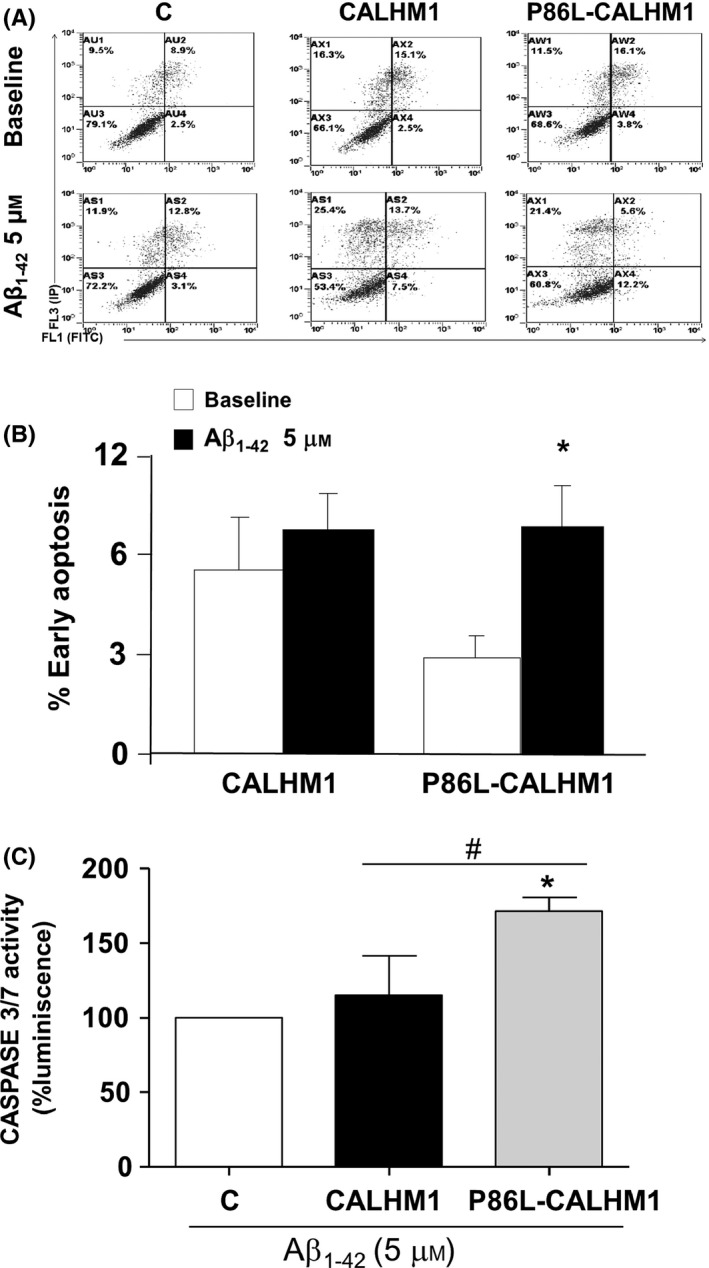
Apoptosis triggered by treatment with Aβ_1–42_ in CALHM1 and P86L‐CALHM1 cells. Determination of the different phases of apoptosis induced by 5 μm Aβ_1–42_ for 24 h in cells transfected with empty vector, CALHM1, and P86L‐CALHM1. (A), a typical flow cytometry; the upper pictures correspond to nontreated control, CALHM1, and P86L‐CALHM1 cells, and the bottom pictures to cells treated with 5 μm of a mixture of protofibrils and oligomers of Aβ_1–42_ for 24 h. (B) shows early apoptotic cells without treatment (white bars) or upon treatment with Aβ (black bars). Pooled data are expressed as mean ± SEM from at least 12 experiments performed with seven different cultures. One‐way *t*‐test, *P < 0.05 compared with nontreated cells. (C), Activation of caspases 3 and 7 induced by Aβ_1–42_. Measurement of activation of caspases 3 and 7 after 8 h of treatment with 5 μm Aβ_1–42_ in control (C), CALHM1‐, and P86L‐CALHM1‐expressing cells. The results are expressed as the difference in relative luminescence units per second after comparing cells in the absence of Aβ. Triplicate measurements were obtained from three different cultures. Data are expressed as mean ± SEM. anova post hoc Tukey, *P < 0.05 compared with control cells. *T*‐test, ^#^ *P* < 0.05 compared with CALHM1 versus P86L‐CALHM1.

To clarify the mechanism involved in the cell death observed in Fig 4B, the next reasonable step was to explore whether treatment with Aβ induced apoptosis in cells overexpressing CALHM1 or P86L‐CALHM1. To this end, we explored the different apoptosis stages in control, CALHM1, and P86L‐CALHM1 cells upon exposure to Aβ_1–42_ (5 μm) for 24 h. We observed a clear tendency toward early triggering of apoptosis only in cells overexpressing the mutated form P86L‐CALHM1 at 3 and 6 h (data not shown). Thus, we incubated the cells overexpressing CALHM1 and P86L‐CALHM1 for a longer time period to determine how long it would the apoptosis stage upon treatment with Aβ. After 24 h, only P86L‐CALHM1‐overexpressing cells activated the early apoptosis pathway (Fig. 5B). These results were independent of cell type, because the neuronal hippocampal cell line HT‐22 overexpressing CALHM1 and P86L‐CALHM1 were also vulnerable when treated with Aβ_25–25_ (50 μm) for 24 h. (Supplemental Results and figures).

To confirm that apoptosis was taking place (observed in Figs. 4B and 5B), we measured caspases 3 and 7. After exposure to Aβ_1–42_ 5 μm for 8 h, the activity of caspases 3 and 7 rose significantly only in cells overexpressing P86L‐CALHM1 versus CALHM1 (Fig. 5C).

### Regulation of ERK and CREB in CALHM1‐ and P86L‐CALHM1‐overexpressing cells

**Figure 6:**
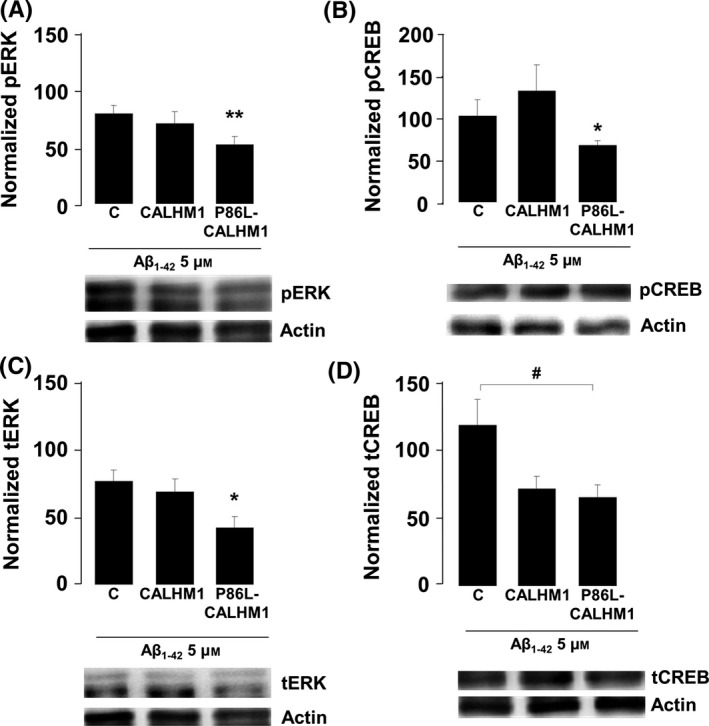
Aβ exposure reduces ERK and CREB signaling in P86L‐CALHM1‐expressing cells. Levels of protein expression of phosphorylated ERK (pERK) (A), total ERK (tERK) (B), phosphorylated CREB (pCREB) (C), and total CREB (tCREB) (D) after 1 h of treatment with 5 μm oligomers of the human fragment Aβ_1–42_ (Aβ_1–42_) in control (C), CALHM1‐ or P86L‐CALHM1‐expressing HeLa cells. Protein levels are normalized with respect to actin and to their respective baseline protein levels in cells without Aβ treatment. Data correspond to the mean ± SEM of five different cultures. anova post hoc Bonferroni, *P < 0.05, **P > 0.01 compared with levels in P86L‐CALHM1 cells not treated with Aβ; anova post hoc Dunnett, ^#^ *P* < 0.05 compared with control cells treated with Aβ_1–42_.

Ca^2+^ is critically involved in synaptic activity and memory formation by regulating specific signal transduction pathways that implicate key protein effectors, such as CAMK, MAPK/ERK, and CREB. Therefore, we performed experiments to clarify whether Aβ‐treated cells expressing CALHM1 or P86L‐CALHM1 could be regulating a key gene implicated in survival pathways such as ERK and CREB. No significant changes were detected in the expression of p‐ERK or t‐ERK between controls and CALHM1 cells that were untreated or treated with Aβ (5 μm of oligomers of Aβ_1–42_ for 1 h). However, treatment with Aβ significantly decreased both p‐ERK expression and t‐ERK expression in P86L‐CALHM1 cells (Fig. 6A and C).

We also measured CREB, a transcriptional factor involved in memory and neuronal survival that can be activated via its phosphorylation at serine (Ser) 133 by several kinases, including ERK. In P86L‐CALHM1 cells treated with Aβ, we observed significantly lower expression of p‐CREB than P86L‐CALHM1 cells not treated with Aβ (Fig. 6B). As for t‐CREB, P86L‐CALHM1 cells treated with Aβ showed significantly lower expression levels than control cells exposed to Aβ; a trend toward lower expression levels was also observed in CALHM1‐expressing cells, although it was not statistically significant (Fig. 6D).

## Discussion

We found alterations in [Ca^2+^]_n_ signaling in HeLa cells transfected with the wild‐type CALHM1 Ca^2+^ channel and its mutated form P86L‐CALHM1. Baseline [Ca^2+^]_n_ values in CALHM1 and P86L‐CALHM1 cells were twice those of controls both after perfusion with a 0 Ca^2+^/EGTA solution and under physiological conditions in 1 mm Ca^2+^ (Figs. 1B and 2B). Furthermore, upon reintroduction of Ca^2+^, the kinetics of the [Ca^2+^]_n_ transients generated developed at a slower rate and decayed to a long‐lasting plateau in CALHM1 and P86L‐CALHM1 compared with control cells (Figs. 1A, C, E). Of interest was the fact that peak and total [Ca^2+^]_n_ elevations (AUC) were somewhat higher in the cells expressing both forms of CALHM1; the differences detected are similar to those observed with [Ca^2+^]_c_ under the same experimental conditions (Moreno‐Ortega *et al*., 2010), indicating that [Ca^2+^]_n_ depends at least partially on [Ca^2+^]_cit_ (Alonso & Garcia‐Sancho, 2011). Except for a slower decay in P86L‐CALHM1 cells, the transients were similar to those of CALHM1 cells. Slower and longer [Ca^2+^]_n_ signals in CALHM1 and P86L‐CALHM1 can be altered because, upon transfection, these channels are preferentially expressed in ER membranes (Dreses‐Werringloer *et al*., 2008) and these membranes form the nuclear envelope; thus, the CALHM1 channel could be eliciting elevation of [Ca^2+^]_n_ by simply acting as a leak channel pore or as an InsP_3_R pore. This last possibility seems unlikely because the [Ca^2+^]_n_ transients generated by histamine were similar in control cells and in CALHM1 and P86L‐CALHM1 cells (Fig. 2). It therefore seems plausible that CALHM1 behaves as a leak Ca^2+^ channel and that the greater baseline [Ca^2+^]_n_ levels in CALHM1 and P86L‐CALHM1 cells could be explained by Ca^2+^ leakage from the ER lumen into the nucleus. This interpretation is in line with the idea that the nucleus might have specific Ca^2+^ transients at specific functionally distinct subcompartments; in fact, the nuclear reticulum can generate localized nuclear Ca^2+^ gradients (Gerasimenko *et al*., 1995). The alternative hypothesis implies that cytosolic Ca^2+^ signals can generate nuclear Ca^2+^ signals by simple Ca^2+^ diffusion (Gerasimenko *et al*., 1995; Chamero *et al*., 2008; Alonso & Garcia‐Sancho, 2011).

Given that the mutated channel P86L‐CALHM1 causes accumulation of Aβ and thus increases Aβ levels in a Ca^2+^‐dependent manner (Dreses‐Werringloer *et al*., 2008), it could be responsible for cell vulnerability. In fact, we observed that expression of P86L‐CALHM1 significantly decreased cell viability (Fig. 4B) by initiating early apoptosis (Fig. 5B) in cells exposed to Aβ compared with treated cells overexpressing the empty vector or the CALHM1 channel. When control, CALHM1‐, or P86L‐CALHM1‐overexpressing cells were exposed to channel activation by Ca^2+^ addback, no significant vulnerability was observed in any of the cells; these results are in agreement with those reported by Dreses‐Werringloer and co‐workers (Dreses‐Werringloer *et al*., 2008). However, mitochondrial oxidative stressor, such as PAO, did not significantly increase the vulnerability of P86L‐CALHM1‐expressing cells compared with control or CALHM1‐expressing cells (Figs. 4C), indicating the presence of a different cell death mechanism between mitochondrial stressors and P86L‐CALHM1‐induced vulnerability. Additionally, the molecular mechanism of cell death implicated in P86L‐CALHM1 cells exposed to Aβ seems to be related to activation of early apoptosis (Fig. 5B); during this phase, phosphatidylserine is flipped to the outer side of the plasma membrane in a caspase‐dependent process (Bouchier‐Hayes *et al*., 2008); therefore, activation of caspases 3 and 7 was observed (see Fig. 5C). It is noteworthy that the cell death mechanism triggered by P86L‐CALHM1 in the presence of Aβ does not depend on the cell type, because the neuronal hippocampal cell line HT‐22 is also vulnerable to this stimulus, which triggers early apoptosis (Fig. S2). Moreover, other AD‐like insults such us okadaic acid (OKA) induced cell death in Hela P86L‐CALHM1‐overexpresing cells versus control and CALHM1, significantly (Fig S3). Taken together, these results could add evidence of the influence of P86L‐CALHM1 on the onset of AD and Aβ.

The enhanced vulnerability of P86L‐CALHM1‐overexpressing cells upon exposure to Aβ has been associated with the reduction observed in the expression of proteins related to cell survival signaling pathways such as ERK and CREB (Walton & Dragunow, 2000; Lonze & Ginty, 2002). In fact, in the presence of Aβ, P86L‐CALHM1‐expressing cells showed lower expression levels of p‐ERK and t‐ERK than controls (Fig. 6A, C). Changes in phosphorylation of ERK in CALHM1 and P86L‐CALHM1 were also recently described by Dreses‐Werringloer *et al*. (2013).

The transcriptional factor CREB has been related to neuronal survival, synaptic plasticity, and memory (Walton & Dragunow, 2000); phosphorylation of its Ser133 has been identified as the key event that must occur for CREB to function as a stimulus‐dependent transcriptional activator. After phosphorylation at Ser133, CREB recruits CREB‐binding protein to act as a transcriptional coactivator (Chrivia *et al*., 1993). A number of Ca^2+^‐dependent signaling pathways, such as MAPK/ERKs, have been implicated in the nuclear phosphorylation of CREB at Ser133. Reduction in t‐ERK and p‐ERK in cells expressing the mutated form of the channel could account for the reduction in p‐CREB detected in these cells (Fig. 6B). Curiously, cells overexpressing CALHM1 tended to reduce t‐CREB values, although this was not related to a diminution in the active form, p‐CREB, or the lack of alterations found in p‐ERK. Therefore, cell vulnerability was not increased.

## Conclusion

We showed that P86L‐CALHM1 impairs plasma and nuclear membrane Ca^2+^ permeability, increases cytosolic and nuclear steady‐state Ca^2+^ levels, depresses the cell survival ERK/CREB pathway, and increases cell vulnerability to Aβ by triggering early apoptosis and activation of caspases 3 and 7. Therefore, in the presence of Aβ, P86L‐CALHM1 seems to shift the balance between neurodegeneration and neuronal survival toward the stimulation of pro‐cytotoxic pathways, which may in turn contribute to its deleterious effects in AD.

## Experimental procedures

### Chemicals

Metafectene^®^ was purchased from Biontex (Munich, Germany). Wild‐type coelenterazine was purchased from Biotium (Hayward, CA, USA). 4′6‐diamidino‐2‐phenylindole (DAPI), Aβ_25–35_, anti‐β‐actin, histamine, human Aβ_1–42_, paraformaldehyde, propidium iodide (PI), thiazolyl blue tetrazolium bromide (MTT), TritonX‐100, and Tween 20 were purchased from Sigma (Madrid, Spain). The antibodies anti‐c‐Myc and anti‐CALHM1 and the PVDF membranes were from Millipore (Madrid, Spain), and Alexa 488 and Image iT‐FX signal enhancer were from Thermo Fisher Scientific (Madrid, Spain). The BCA Protein Assay Kit Reagent was from GE^™^ Healthcare, and the ECL Advance^™^ Western Blotting Detection Kit was from Fisher Scientific. The DAKO^®^ mounting medium was purchased from DAKO (Barcelona, Spain). The antibodies anti‐CREB, anti‐P‐CREB, and anti‐P‐ERK were from Cell Signalling (Madrid, Spain); anti‐total ERK and secondary antibodies were purchased from Santa Cruz Biotechnology (Santa Cruz, USA). We analyzed apoptosis using the FITC Apoptosis Detection Kit (Immunostep, Salamanca, Spain). Other general chemicals were purchased from Sigma (Madrid, Spain) or Panreac Química S.L.U. (Barcelona, Spain). The cDNA encoding for aequorins was a gift from Professor Tullio Pozzan (University of Padua). The cDNA encoding for CALHM1 and P86L‐CALHM1 was a gift from Professor Philippe Marambaud (Albert Einstein College of Medicine, New York, USA).

### Culture of HeLa cells

HeLa cells were grown in plastic flasks in DMEM supplemented with 10% fetal bovine serum, 2 mm glutamine, 25 U mL^−1^ penicillin, and 25 μg mL^−1^ streptomycin (all products purchased from Lonza, Basel, Switzerland).

### Measurements of [Ca^2+^]_n_ and [Ca^2+^]_c_ with aequorins

Cell experiments were performed with 8 × 10^4^ cells seeded on 12‐mm‐diameter coverslips and grown to 60–70% confluence. Transfection with the genetically encoded photoprotein aequorins targeting the nucleus (nu_AEQ) or cytosol (cyt_AEQ) was achieved using Metafectene^®^ as described elsewhere for cells (Moreno‐Ortega *et al*., 2010). Empty vector (control), or vectors containing CALHM1 or P86L‐CALHM1 were transiently co‐transfected with aequorins at a ratio of 1:1. Experiments to measure changes in [Ca^2+^]_n_ or [Ca^2+^]_c_ were performed 36 to 48 h after transfection. The two recombinant proteins were expressed in the same subset of cells (AEQ and the channel) (Brini *et al*., 1995).

HeLa cells expressing nu_AEQ or cyt_AEQ were reconstituted by adding 5 μm wild‐type coelenterazine for 1.5 h before the experiment. To ensure the total translocation of nu_AEQ to the nucleus, cells were incubated with dexamethasone 10 μm for 2 h immediately before the experiment was carried out (Brini *et al*., 1995). The cell monolayer was continuously superfused at room temperature (24 ± 2 °C) with Krebs–Hepes buffer (KHB) of the following composition: 125 mm NaCl, 5 mm KCl, 1 mm Na_3_PO_4_, 1 mm MgSO_4_, 5.5 mm glucose, and 20 mm HEPES (pH 7.4); the zero Ca^2+^ solution contained 0.5 mm ethylene glycol tetraacetic acid. To induce entry of Ca^2+^, KHB deprived of Ca^2+^ was switched to another solution containing 1 mm CaCl_2_, as specified in the figure legends. When used, 10 μm Aβ_25–35_ or 100 μm histamine was added to the KHB. Light emission was measured in a purpose‐built luminometer and calibrated in terms of [Ca^2+^], as described by (Rizzuto *et al*., 1992). At the end of the experiment, cells were lysed by superfusing them with KHB containing 10 mm CaCl_2_ and 100 μm digitonin to expose them to excess Ca^2+^ to burn out the aequorin remaining at the end of each experiment and to normalize the Ca^2+^ transients to the fraction of total aequorin consumed at each point during the experiment.

### Cell treatment with a mixture of protofibrils and oligomers of Aβ_1–42_

HeLa cells were seeded on 24‐well plates and transfected as described above; 24 h after transfection, cells were treated with a mixture of protofibrils and oligomers of Aβ_1–42_ (Aβ_1–42_) (5 μm) for 1 h and then harvested and lysed to determine the expression of CREB, pSer133CREB, ERK1/2, and pERK1/2 (Western blot).

Aggregation of Aβ_1–42_ at 5 μm was achieved as previously described (Parodi *et al*., 2010). Briefly, human Aβ_1–42_ was dissolved with dimethylsulfoxide (DMSO) at 2.3 mm; an aliquot of the 2.3‐mm solution was then dissolved in PBS to a final concentration of 80 μm. Finally, this solution was incubated at 37 °C for 2 h under constant shaking.

### Monitoring of cell viability

Cell viability was measured using an MTT assay as described elsewhere (Alonso *et al*., 2013). Briefly, 5 × 10^4^ HeLa cells were seeded in 48‐well plates and transfected with 0.5 μg of empty vector, CALHM1, or P86L‐CALHM1. Twenty‐four hours after transfection, cells were treated with 5 μm of aggregated Aβ_1–42_, oligomycin (10 μm) plus rotenone (30 μm), or phenylarsine oxide (PAO, 1 μm) for 24 h. Cells were then incubated with MTT reagent for reducing for 30 min to form formazan crystal, which was then dissolved with DMSO. Optical density (OD) was read using an ELISA reader at 540 nm (Berthold Detection Systems, Sirius). Cell viability was expressed as a percentage of the control and calculated using the following equation: viability = [OD test/OD baseline] × 100.

### Analysis of apoptosis phases

We analyzed the different phases of apoptosis using the FITC Apoptosis Detection Kit (Immunostep, Salamanca, Spain), which is based on the ability of annexin V to bind specifically to phosphatidylserine flipped into the outer layer of the plasma membrane and the ability of the nonvital dye propidium iodide (PI) to bind to DNA only in altered membrane cells. Thus, double staining enables discrimination between intact cells (annexin V–negative and PI‐negative), early apoptotic cells (annexin V–positive, PI‐negative), and late apoptotic cells (annexin V‐positive and PI‐positive) or necrotic cells (without the characteristic cell integrity).

In brief, HeLa (2 × 10^5^) cells were seeded in 6‐well plates and transfected with 0.75 μg of empty vector and 2 μg of CALHM1 or P86L‐CALHM1. Twenty‐four hours after transfection, HeLa cells were treated for 24 h with 5 μm of a mixture of protofibrils and oligomers of Aβ_1–42_ and collected and incubated with fluorescent annexin V and PI. Apoptosis was determined by flow cytometry.

### Measurement of caspase activation

We measured the activation of caspases 3 and 7 using a commercial kit based on luminescence (Caspase‐Glo (R) 3/7 Assay, Promega Biotech Ibérica S.L., Madrid, Spain) as described by (Alonso *et al*., 2013). In brief, 10^5^ HeLa cells were seeded in 48‐well plates and transfected with 0.5 μg of empty vector, CALHM1, or P86L‐CALHM1. Twenty‐four hours after transfection, cells were treated with 5 μm of a mixture of protofibrils and oligomers of Aβ_1–42_ for 8 h. The readings were performed in black 96‐well plates with a plate reader (Glo‐Max Multi Detection System, Promega Biotech Ibérica S.L., Madrid, Spain). Activation of caspases 3 and 7 is expressed as a percentage of nontreated transfected cells.

### Measurement of protein expression by Western blot

HeLa cells transfected with 0.5 μg of empty vector, CALHM1, or P86L‐CALHM1 and treated or not with 5 μm of a mixture of protofibrils and oligomers of Aβ_1–42_ for 24 h were lysed with 100 μL of cold lysis buffer containing 1% Nonidet P‐40, 10% glycerol, 137 mm NaCl, 20 mm Tris‐HCl pH 7.5, 1 mg mL^−1^ leupeptin, 1 mm phenylmethylsulfonyl fluoride, 20 mm NaF, 1 mm Na_4_P_2_O_7_, and 1 mm Na_3_PO_4_. Once the amount of protein was quantified using the BCA Protein Assay Kit reagent, electrophoresis was performed by running 30 μg of protein in polyacrylamide gels for 2 h at constant amperage. Proteins were transferred to PVDF membranes for 2 h at 70 mA. Membranes were then blocked for 2 h with Tween 20‐Tris Buffered Saline (TTBS) containing albumin 4% and incubated with anti‐p‐ERK, anti‐total ERK, anti‐P‐CREB, or anti‐total CREB and anti‐β actin for 2 h. After washing several times with TTBS, the corresponding secondary antibodies were added for 45 min. Finally, the membranes were revealed using the Western Blotting Detection Kit (Thermo Fisher Science) and analyzed and quantified using Scion‐Image software (Scion Corporation Informer Technologies Inc, Meyer Instruments Inc., Houston, EEUU).

### Statistics

Values are given as mean ± SEM. The statistical differences between means were assessed using the *t*‐test or anova and Bonferroni's, Dunnett's, or Tukey's tests in a post hoc analysis. Differences between experimental groups were considered statistically significant at *P* < 0.05.

## Funding

This work was partly supported by the following grants: Ministerio de Economía y Competitividad, FPU Program, Refs. AP2009/0343 (AJMO) and AP2010/1219 (IB). ARN: FIS PI10/01426. MGL: Ministerio de Economía y Competitividad, Ref. SAF2012‐23332. MFCA: Consolidación de grupos de investigación UAM‐CAM 1004040047. We also thank Fundación Teófilo Hernando, Madrid, Spain, for their continued support.

## Conflict of interest

None declared.

## Supporting information


**Fig. S1** Cellular localization of CALHM1 and P86L‐CALHM1.

**Fig. S2** Cell vulnerability after treatment with Aβ_25–35_ in HT‐22.

**Fig. S3** Early apoptosis triggered by treatment with Aβ_25–35_ in CALHM1‐ and P86L‐CALHM1–expressing HT‐22 cells.

**Fig. S4** Dose response curve of Okadaic Acid.

**Data S1** Supplemental Material and Methods.
