# Supplementary figures and images for: CALHM1 and its polymorphism P86L differentially control Ca2+ homeostasis, mitogen‐activated protein kinase signaling, and cell vulnerability upon exposure to amyloid β
Source: Aging Cell. 2015 Sep 29;14(6):1094–102. doi: 10.1111/acel.12403 (PMC4693463; doi:10.1111/acel.12403)

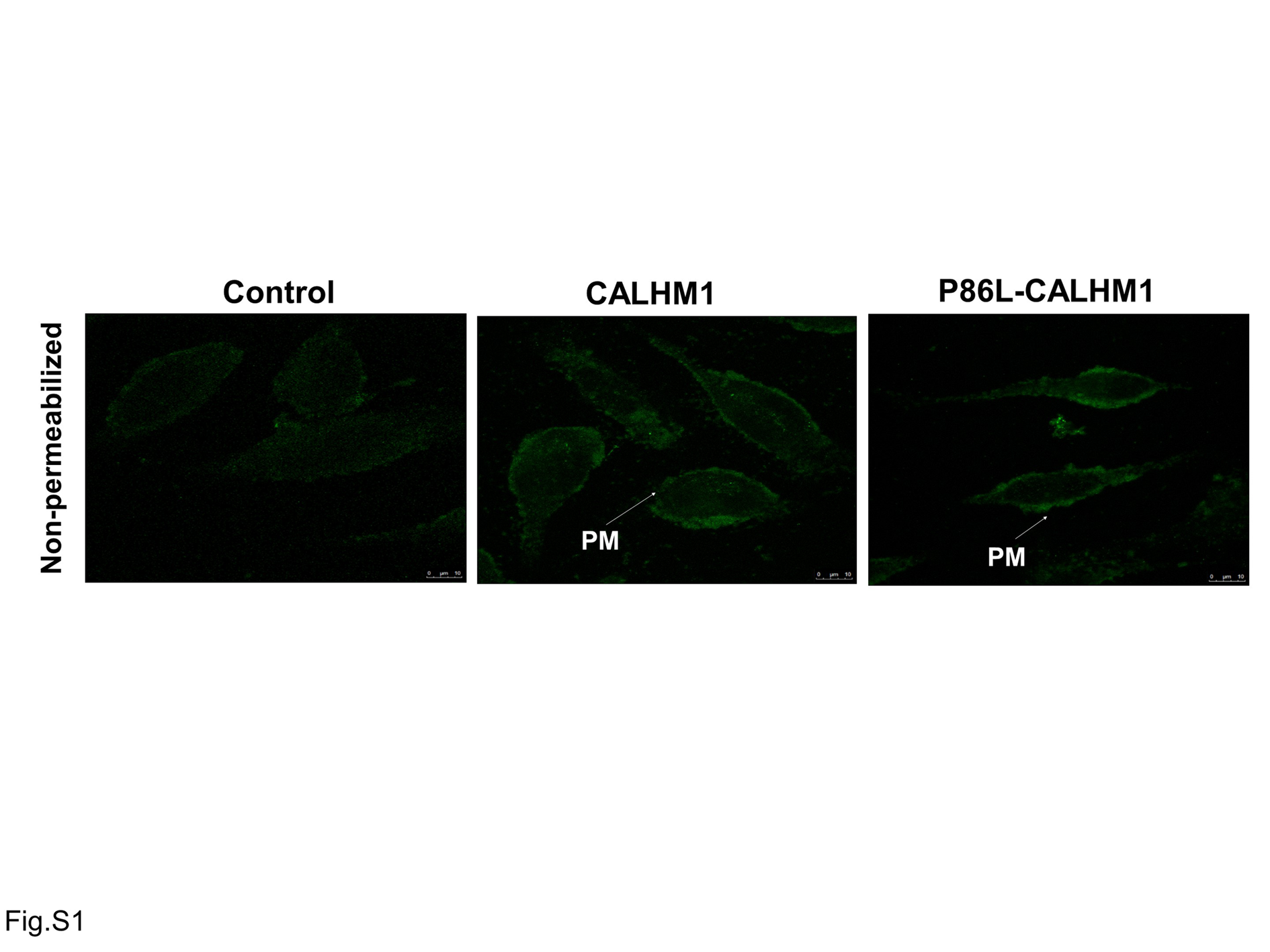

Supplement: Supplementary file 1 — Fig. S1 Cellular localization of CALHM1 and P86L‐CALHM1. [file ACEL-14-1094-s001.tif]

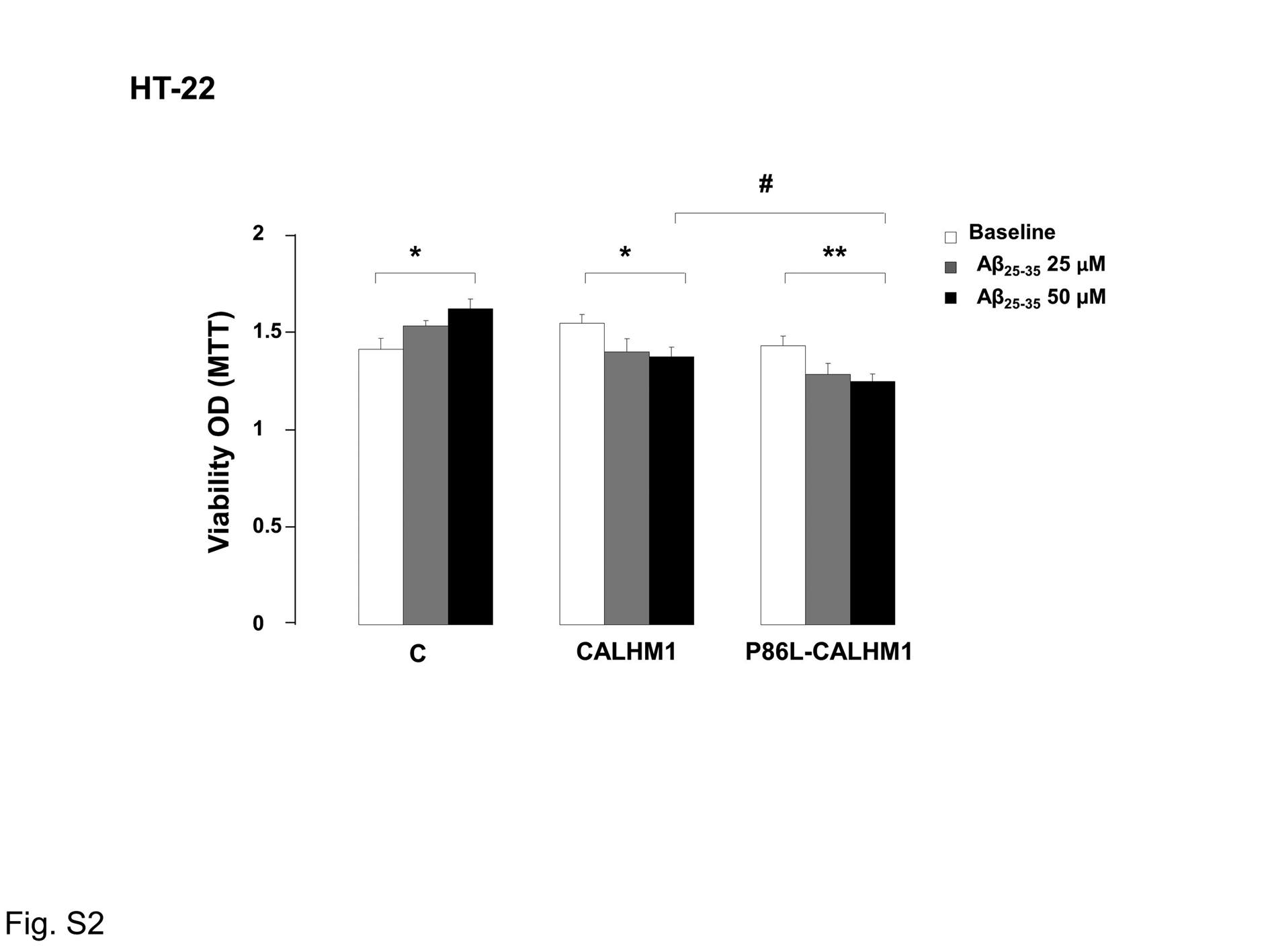

Supplement: Supplementary file 2 — Fig. S2 Cell vulnerability after treatment with Aβ25–35 in HT‐22. [file ACEL-14-1094-s002.tif]

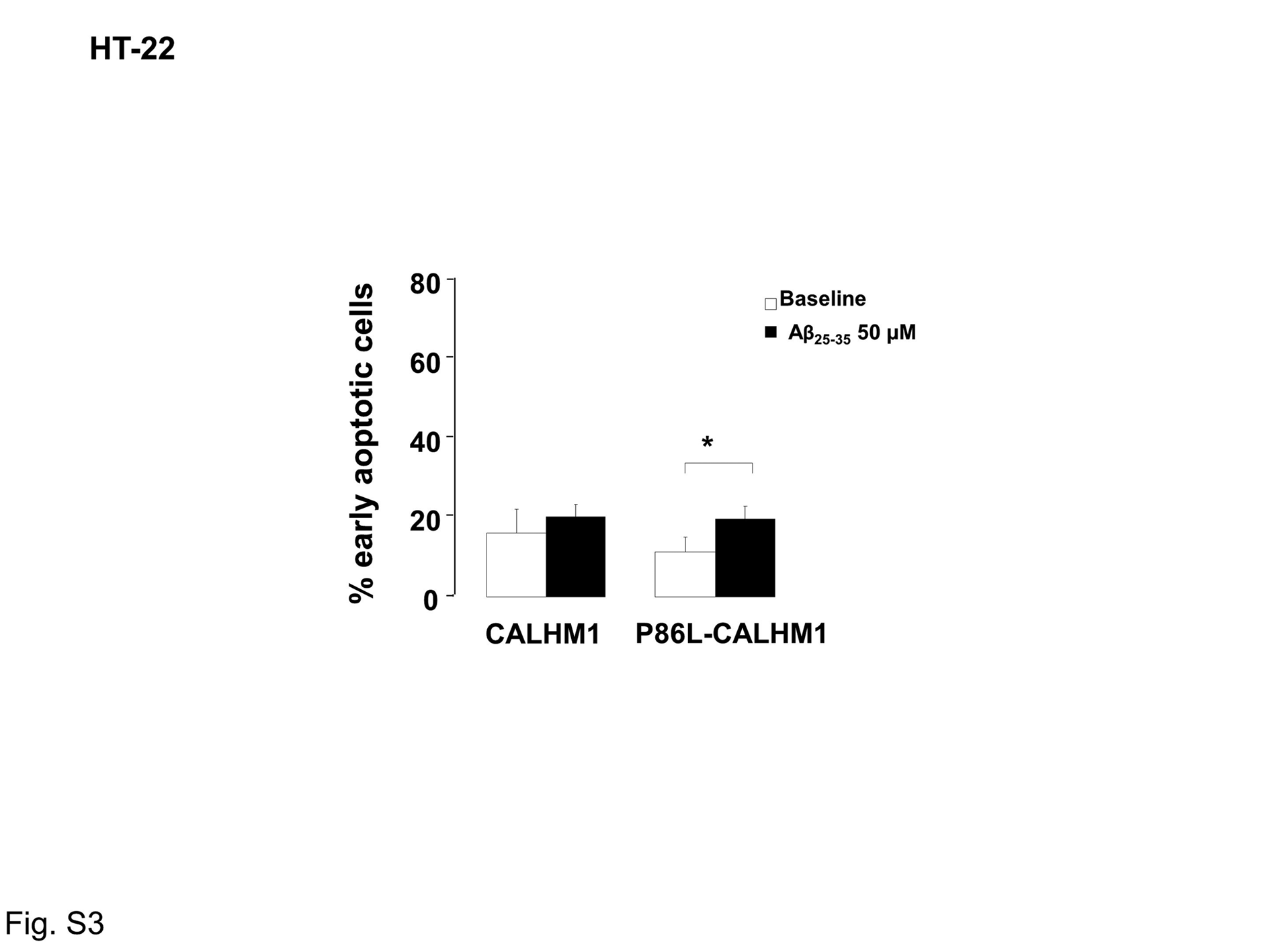

Supplement: Supplementary file 3 — Fig. S3 Early apoptosis triggered by treatment with Aβ25–35 in CALHM1‐ and P86L‐CALHM1–expressing HT‐22 cells. [file ACEL-14-1094-s003.tif]

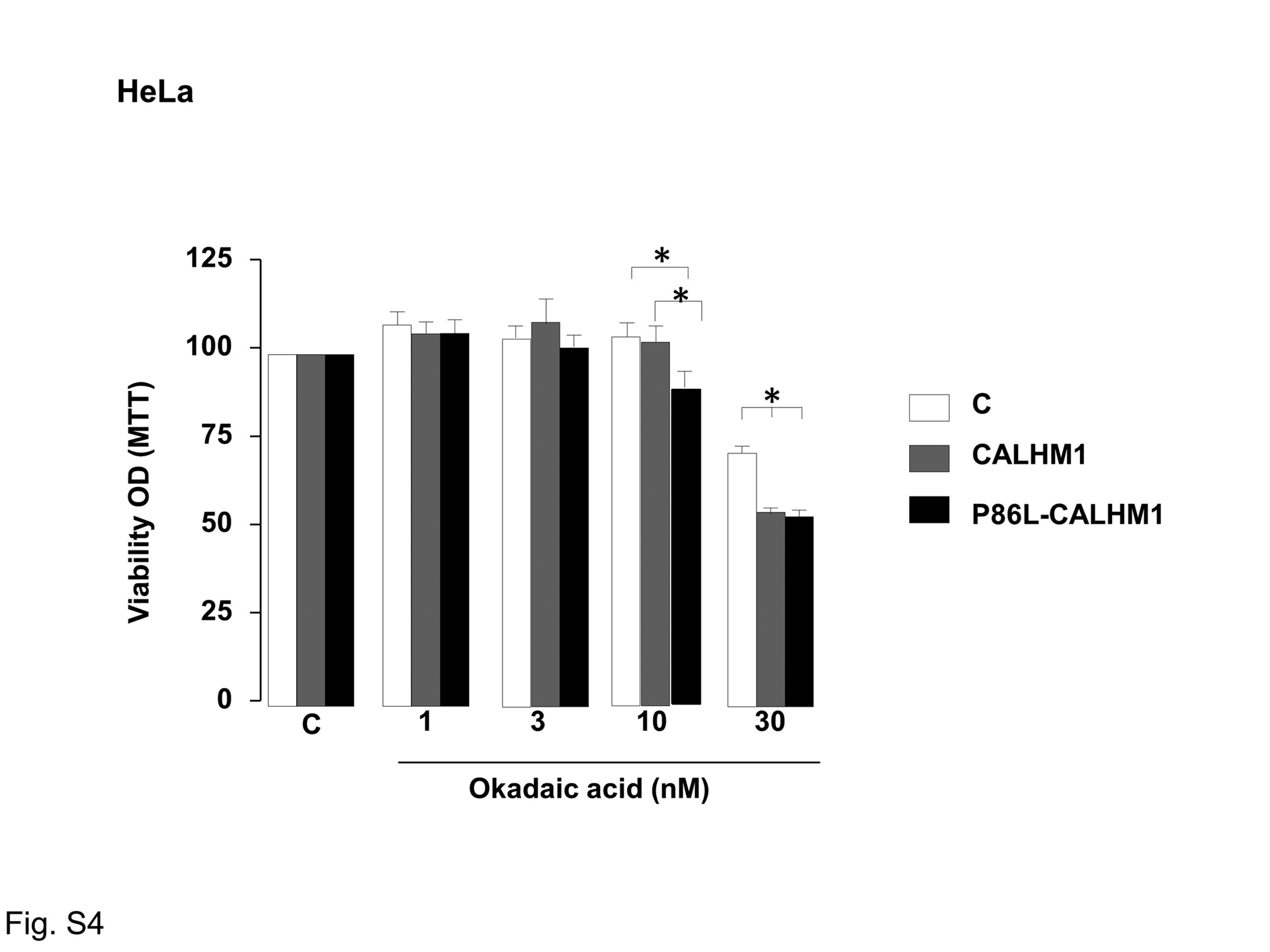

Supplement: Supplementary file 4 — Fig. S4 Dose response curve of Okadaic Acid. [file ACEL-14-1094-s004.tif]
