# Supplementary material for: CALHM1 and its polymorphism P86L differentially control Ca2+ homeostasis, mitogen‐activated protein kinase signaling, and cell vulnerability upon exposure to amyloid β
Source: Aging Cell. 2015 Sep 29;14(6):1094–102. doi: 10.1111/acel.12403 (PMC4693463; doi:10.1111/acel.12403)
Supplement: Supplementary file 5 — Data S1 Supplemental Material and Methods. [file ACEL-14-1094-s005.pdf]

Supplementary documents

Supplemental Material and Methods

Immunofluorescence analysis and confocal microscopy

HeLa cells were grown at a density of $6x10^4$ on 12-mm coverslips and transfected with empty vector (control), CALHM1, or P86L-CALHM1 using Metafectene®. Briefly, immunostaining was performed in cells washed with phosphate-buffered saline (PBS), fixed with 4% paraformaldehyde for 20 min and, if necessary, permeabilized with 0.1% TritonX-100 in PBS for 5 min. The fixed cells were rehydrated with PBS and blocked with Image-iT FX signal enhancer for 30 min. Non-permeabilized cells were then incubated with anti-CALHM1 (1:100) ~~and permeabilized cells were incubated with anti-Myc (1:100)~~ as primary antibody~~ies~~ for 45 min. After washing, the cells were incubated with Alexa 488 (1:1000) for 45 min. At the end of incubation, the nuclei were stained using 1 µg/mL DAPI for 10 min. Finally, cells were rinsed in PBS and mounted in DAKO® medium. Images were acquired using a Leica TCS-SP5 confocal microscope (Leica Microsystems, Barcelona, Spain).

Culture of HT-22 cells

HT-22 cells were grown in plastic flasks in DMEM supplemented with 10% fetal bovine serum, 2 mM glutamine, 25 U/ml penicillin, and 25 µg/ml streptomycin (all products purchased from Lonza, Basel, Switzerland).

Monitoring of cell viability

Cell viability was measured using the MTT assay as described elsewhere (Alonso *et al.* 2013). Briefly, $5x10^4$ HT-22 cells were seeded in 48-well poly-L-lysine–coated plates and transfected with 0.5 µg of empty vector, CALHM1, or P86L-CALHM1 using Metafectene©. Twenty-four hours after transfection, cells were treated with 25-50 µM $A\beta_{25\text{-}35}$ for 24 h and then incubated with MTT reagent for reducing for

30 min to form formazan crystal, which was dissolved with DMSO. Optical density (OD) was read using an ELISA reader at 540 nm (Berthold Detection Systems, Sirius). Cell viability was estimated as OD.

Analysis of apoptosis phases

We analyzed the different phases of apoptosis using the FITC Apoptosis Detection Kit (Immunostep, Salamanca, Spain), which is based on the ability of annexin V to bind specifically to phosphatidylserine flipped into the outer layer of the plasma membrane and the ability of the non-vital dye propidium iodide (PI) to bind to DNA only in altered membrane cells. Thus, double staining makes it possible to distinguish between intact cells (annexin V–negative and PI-negative), early apoptotic cells (annexin V–positive, PI-negative), and late apoptotic cells (annexin V–positive and PI-positive) or necrotic cells (without the characteristic cell integrity).

In brief, HT-22 cells ($5x10^5$) were seeded in 6-well poly-L-lysine–coated plates and transfected with 1 μg of empty vector, CALHM1, or P86L-CALHM1. Twenty-four hours after transfection, HT-22 cells were treated with $A\beta_{25-35}$ (50 μM) for 12 h. They were then collected and incubated with fluorescent annexin V and PI. Apoptosis was determined by flow cytometry.

Supplemental Results

Characterization of CALHM1 and P86L-CALHM1 overexpressing in HeLa cells

Immunostaining was carried out under non-permeabilizing conditions (**Fig. S1A**) ~~and permeabilizing conditions (**Fig. S1B**) to verify the distribution of~~ for localization of CALHM1 and P86L-CALHM1 overexpressed in HeLa cells. CALHM1 was evenly distributed in the plasma membrane in non-permeabilized cells (**Fig. S1~~A~~**). ~~However, under permeabilizing conditions (**Fig. S1B**), the ER distribution of CALHM1 was reproduced as described by (Dreses-Werringloer *et al.* 2008).~~ The distribution and location of P86L-CALHM1, as expected, was similar to that of native CALHM1 ~~(**Figs. S1A and B**).~~

Dose response curve of Okadaic Acid. P86L-CALHM1 overexpression renders more vulnerable cells against okadaic acid

Okadaic acid (OKA) is associated with protein phosphorylation; it is implicated in hyperphosphorylation of tau and in later stages causes AD-like pathology. In order to evaluate, another insult related with AD, OKA was tested in Hela cells overexpressing CALHM1 and P86L-CAHM1. A dose response curve was performed 1, 2, 10 and 30 nM for 24 hours. At 1 and 3 nM okadaic acid was not cytotoxic for any cell type. However, OKA at 10 nM, induced a significant cytotoxic effect on P86L-CALHM1 overexpressing cells, ~~. It was~~ 10.51% of cell death versus, ~~meaning more vulnerable than~~ control ~~cells~~ and CALHM1 overexpressing ones; those finding show~~s~~ how P86L-CALHM1 selectively induces increased vulnerability to toxic stimuli related to AD. Meanwhile, ~~administration~~ incubation ~~of~~ with OKA, 30 nM for 24 h, reduced cell viability ~~decreased~~ around 28.1% in control cells, while ~~the cells transfected with the~~

~~channel such reduction was~~ for CALHM1 and P86L-CALHM1 overexpressing ones, cell death was 44.82% ~~(for CALHM1)~~ and 45, 66% ~~(for P86L-CALHM1)~~ respectively (**Fig. 4S**)

Supplemental Figure Legends

Figure S1

Cellular localization of CALHM1 and P86L-CALHM1

Immunostaining of CALHM1 (in green) in HeLa cells transfected with pcDNA3.1 (Control), CALHM1 or P86L-CALHM1. The nucleus has been stained with DAPI. **(A)** Immunofluorescent staining under non-permeabilizing conditions. ~~**(B)** Immunofluorescent staining under permeabilizing conditions.~~

Figure 2S

Cell vulnerability after treatment with A$\beta_{25\text{-}35}$ in HT-22

The MTT assay was performed to test cell viability in control (C), CALHM1-, or P86L-CALHM1–expressing HT-22 cells. Viability was determined after 12 h of treatment with 25-50 µM of fragment 25-35 of Aβ (A$\beta_{25\text{-}35}$).

White bars represent non-treated cells, while grey and black bars correspond to cells treated with 25 and 50 µM of A$\beta_{25\text{-}35}$, respectively, for 12 h. Triplicate measurements were obtained from 3 different cultures. Data are expressed as the mean±SEM, *t* test. *$p<0.05$ compared to non-treated cells. #$p<0.05$ comparing CALHM1- and P86L-CALHM1–expressing cells treated with 50 µM of A$\beta_{25\text{-}35}$.

Figure 3S

Early apoptosis triggered by treatment with Aβ_{25-35} in CALHM1- and P86L-CALHM1–expressing HT-22 cells

Determination of the early apoptosis induced by 50 μM of Aβ_{25-35} for 12 h as a percentage of annexin V–positive cells in control (C), CALHM1-, and P86L-CALHM1–expressing HT-22 cells. Pooled data are expressed as mean±SEM from 3 different cultures. *t* test,*$p<0.05$ compared with non-treated cells.

Figure 4S

Dose response curve of Okadaic Acid. P86L-CALHM1 renders more vulnerable cells against okadaic acid

The MTT assay was performed to test cell viability in control (C), CALHM1, or P86L-CALHM1–expressing Hela cells. Viability was determined after 18 h of treatment with 1, 3, 10 and 30 nM of Okadaic acid. Data from four cultures (in triplicate). Differences analyzed with ANOVA and Bonferroni as post-analysis, with * = $p <0.05$.
